# Association of Age-Related Trends in Blood Pressure and Body Composition Indices in Healthy Adults

**DOI:** 10.3389/fphys.2018.01574

**Published:** 2018-11-26

**Authors:** Wei Li, Yan He, Lili Xia, Xinghua Yang, Feng Liu, Jingang Ma, Zhiping Hu, Yajun Li, Dongxue Li, Jiajia Jiang, Guangliang Shan, Changlong Li

**Affiliations:** ^1^Department of Epidemiology and Biostatistics, School of Public Health, Capital Medical University, Beijing, China; ^2^Municipal Key Laboratory of Clinical Epidemiology, Beijing, China; ^3^Shanxi Provincial Disease Prevention and Control Center, Xi’an, China; ^4^Institute of Basic Medical Sciences, Chinese Academy of Medical Sciences, Beijing, China; ^5^School of Basic Medicine, Capital Medical University, Beijing, China

**Keywords:** adiposity, body composition, blood pressure, muscle mass, aging

## Abstract

**Purpose:** Adiposity is one of the important determinants of blood pressure. The aim of this study is to evaluate the association between blood pressure and body composition indices throughout the whole lifespan of healthy adults.

**Patients and Methods:** This study was from an ongoing cross-sectional survey of the Chinese health wherein data included basic physiological parameters. Partial Pearson correlation analysis was used to assess the correlation between blood pressure and body composition indices. Multiple linear regression analysis was used to assess the association of body mass index, lean mass percent, and visceral fat rating against blood pressure in each age group.

**Results:** In the whole population stratified by gender, while body mass index had the highest r-value of correlation with systolic blood pressure in both males (*r* = 0.296, *p* < 0.001) and females(*r* = 0.237, *p* < 0.001), and fat mass percent had the highest *r*-value of correlation with diastolic blood pressure in males (*r* = 0.351, *p* < 0.001) and females(*r* = 0.277, *p* < 0.001), the strength of association with blood pressure were similar across most of the body composition indices. In multiple linear regression analysis, both body mass index and visceral fat rating were positively while lean mass percent was negatively associated with blood pressure in all age groups in both genders, whereas all the association was weaker in the elderly compared to the younger.

**Conclusion:** Maintain the total body fat in a favorable range and appropriately increase the body muscle mass is a strategy to reduce the occurrence of cardiovascular event by decreasing the risk of hypertension through the whole adult life.

## Introduction

China has been undergoing major transitions in health trends particularly toward major increases in chronic non-communicable diseases, such as cardiovascular disease (CVD). Among the CVD risk factors, age is considered as the most important predictor of CVD events (Wald et al., 2011) and hypertension is a major cause of CVD mortality (Ishizaka et al., 2009; Timpson et al., 2009). Age-related increase in blood pressure (BP) is recognized as a universal feature of human aging (O’Rourke and Nichols, 2005; Baksi et al., 2009). Previous epidemiological surveys have shown a progressive increase in systolic blood pressure (SBP) with age, whereas diastolic blood pressure (DBP) also initially increases with age but falls at latter ages (Franklin et al., 1997). It has been reported that even a decline of SBP at baseline by 2 mmHg is related to a 5% reduction of 16-year mortality from CVD (Ikeda et al., 2008).

Thus, effective control of BP is essential for improving population health. Mendelian randomization studies of BP associated with adiposity-related genetic variants (Timpson et al., 2009; Holmes et al., 2014) and controlled trials of weight loss interventions (Cutler, 1991; Stevens et al., 2001) have established the causal relationship between adiposity and BP. Regardless of age and other unmodifiable CVD risk factors such as sex and race, there are many risk factors that are manageable and can be controlled through lifestyle modification, including reduction of obesity (Stoner et al., 2012).

However, there are inconsistencies as to whether a general or central adiposity is more strongly associated with BP and different opinions about which variable is the strongest predictor of BP (Lee et al., 2008; Tuan et al., 2010; Chakraborty and Bose, 2012; Chen Z. et al., 2015). A cross-sectional study in Chinese found that BP was more strongly associated with general adiposity (Chen Z. et al., 2015), while a meta-analysis including ten studies concluded that the measurement of central obesity provided a superior tool for cardiovascular risk (Lee et al., 2008). Understanding the factors affecting age-related BP increase is of obvious clinical importance. Also, to the best of our knowledge, information remains equivocal regarding the strength of the association between body composition indices and BP throughout the whole adult life span in the healthy population.

In consideration of the above, the present study aimed to investigate how BP and body composition change within different age groups and their correlation across the adult age span. We also investigated the contribution of body composition measures (including body mass index (BMI), lean mass percent (LM%), and visceral fat rating (VFR) to the age-related alteration of BP across ten 5-year age groups ranging from 18–79 years in a sample of healthy Chinese adults.

## Materials and Methods

### Ethical Statements

All procedures performed in studies involving human participants were in accordance with the ethical standards of the institutional and/or national research committee and with the 1964 Helsinki declaration and its later amendments or comparable ethical standards. This article does not contain any studies with animals performed by any of the authors. This study was approved by the Institutional Review Board of the Institute of Basic Medical Sciences, Chinese Academy of Medical Sciences. Written consent was obtained from each participant before data collection.

### Study Design and Population

An ongoing population-based cross-sectional survey of Chinese people encompassing health and basic physiological parameters was conducted from 2013 onward, which covered five provinces, including Hainan, Shanxi, Qinghai, Gansu, and Jiangxi.

The current study is based on the data derived from several sites in the Shanxi province, which included 6410 participants. These participants were selected from two urban (Xi’an City and Hanzhong City) and two rural (Qishan County and Hu County) areas which were randomly sampled from the 104 districts in the Shanxi province. In those four regions, we chose six residential communities and six natural villages randomly, based on the list provided by the Center for Disease Control of the Shanxi province (Xia et al., 2017). Pregnant women were not allowed to participate in this study. All subjects, enrolled between June 2014 and July 2014, were required to complete a detailed questionnaire with information of demography, history of disease and corresponding medication use, smoking status and alcohol consumption, and they all underwent a physical examination. Among the 6410 participants, 559 subjects younger than 18 years and 1763 participants with pre-existing hypertension or systemic diseases were excluded from the study. Finally, 4088 healthy participants were included.

### Data Collection

A previously validated questionnaire included the information of demography, history of disease and corresponding medication use, smoking status, and alcohol consumption was administered by the trained technical staff. Awareness of hypertension was determined by asking the subjects if they had been told by a medical doctor that they had hypertension or elevated BP. Antihypertensive medication treatment was determined by asking the subjects if they were currently taking any antihypertensive drugs and checking the drugs they were using.

### Anthropometric Measurements

Anthropometric measurements, including height, weight, waist circumference (WC), and hip circumference (HC) were measured. All of these measurements were made at one time point by trained technicians. Height was measured barefoot using a wall-mounted stadiometer to the nearest 0.1 cm. WC was measured by using a measuring tape at the midpoint between the last floating rib and the top of the iliac crest in the midaxillary line at the end of a gentle expiration. HC was measured by using a measuring tape at the maximum extension of the buttocks. Waist-to-hip ratio (WHR) was calculated as WC (cm) divided by HC (cm). Waist-to-height ratio (WHtR) was calculated as WC (cm) divided by height (cm). Blood pressure of the subject was examined by specialized technical staff in the clinic office in the morning. An electronic sphygmomanometer (HEM-7000, OMRON, JD Hoofddorp, Netherlands), which has been previously validated according to the British and Irish Hypertension Society (BIHS), was used to record SBP and DBP following a resting period of at least 10 min (Belghazi et al., 2007). The subject’s arm was placed at the level of the heart, and BP status was based on the average of three measurements. The estimated values for fat mass, lean mass and visceral fat were provided by using a single frequency (50 kHz) leg-to-leg BIA machine (Model Tanita BC-420MA, Tanita Corporation, Tokyo, Japan) according to the manufacturer’s protocols (Tanita Corporation). All the participants were measured at wearing light clothing and bare feet in a state of fasting and emptying their bladders in the morning. After entering the basic information (ID number, name, gender, birth date, height) of the participant, chose the appropriate model of ordinary people or athletes and then started to test. The estimated weight of cloths was subtracted by 0.5 kg. The analysis was carried out using the software package R (V.1.00). BMI was calculated as weight (kg)/height (m)^2^, FM% and LM% were calculated as fat mass (kg) or lean mass (kg) divided by weight (kg), respectively. VFR rating from 1 to 9 indicated that the participant had a healthy level of visceral fat, and rating from 10 or above indicated an excess level of visceral fat.

### Definitions of Hypertension and Systemic Disease

Hypertension was defined as mean systolic BP (SBP) ≥ 140 mmHg or mean diastolic BP (DBP) ≥ 90 mmHg (Mangat et al., 2015). In this study, elderly was defined as people with age of 60 years or above, and systemic disease was defined by the clinicians as major systemic disorders of the heart, kidney, liver, lung, thyroid, parathyroid, adrenal glands, digestive system disease, connective tissue diseases, hematological diseases, endocrine system diseases, nervous system disease, or a history of malignant tumors.

### Statistical Analysis

All analyses were performed using the SPSS version 19.0 (IBM Corp, Chicago, IL, United States). Graphs were created using R version 3.5.1^1^. The data was expressed as mean ± standard deviation (SD) for continuous variables and number (percentage) for categorical variables. Students’*t*-test and Chi-square analysis was used for describing the differences of continuous variables and categorical variables between males and females Analysis of one-way ANOVA was used to evaluate the difference among age groups. Partial Pearson correlation coefficients were used to assess the relation between body composition indices and BP in all the subjects in the study. Multiple linear regression analysis was employed to evaluate the association of body composition indices and BP in each age group, in which residential location, education level, smoking status and alcohol consumption were adjusted as potential confounding factors. *P*-value < 0.05 (two-tailed) was taken as statistically significant.

## Results

### General Characteristics of the Participants

The general characteristics of the 4088 participants stratified by gender are shown in Table 1. Mean age was 43 (43 ± 13) years old. 59.2% (2419/4088) were females. All of the anthropometric measurements differed significantly by gender except for WHtR after adjustment for age. Height, weight, WC, HC, BMI, WHR, LM%, and VFR were higher in males, while fat mass percent (FM%) was higher in females. As for BP, both SBP and DBP were also higher in males. Male participants had a higher level of education status and a larger proportion was current-smokers and current- alcohol drinkers.

**Table 1:** Characterisitics of the participants.

	All participants (*n* = 4088)	Male(*n* = 1669)	Female (*n* = 2419)	*P*
Age(years)	43.23 ± 13.39	44.07 ± 13.85	42.66 ± 13.04	0.001
Height (cm)	162.00 ± 8.22	169.10 ± 6.08	157.07 ± 5.44	<0.001
Weight (kg)	60.86 ± 11.18	67.51 ± 10.90	56.26 ± 8.81	<0.001
BMI (kg/m^2^)	23.12 ± 3.41	23.57 ± 3.36	22.81 ± 3.40	<0.001
WC(cm)	82.92 ± 10.52	87.08 ± 9.85	80.10 ± 10.01	<0.001
HC(cm)	95.24 ± 6.20	97.19 ± 5.69	93.91 ± 6.18	<0.001
WHR	0.87 ± 0.07	0.89 ± 0.07	0.85 ± 0.07	<0.001
WHtR	0.51 ± 0.06	0.51 ± 0.06	0.50 ± 0.07	0.346
FM (kg)	16.07 ± 6.32	13.82 ± 5.80	17.63 ± 6.19	<0.001
FM%	26.10 ± 7.97	19.73 ± 5.71	30.52 ± 6.11	<0.001
LM(kg)	42.35 ± 8.34	50.91 ± 5.64	36.42 ± 3.14	<0.001
LM%	69.85 ± 7.67	76.09 ± 5.41	65.53 ± 5.84	<0.001
VFR	7.09 ± 4.00	9.68 ± 4.36	5.30 ± 2.45	<0.001
SBP (mm Hg)	116.03 ± 14.50	120.72 ± 12.95	112.79 ± 14.62	<0.001
DBP (mm Hg)	73.32 ± 9.80	76.15 ± 9.68	71.36 ± 9.40	<0.001
Location				0.007
Hanzhong City	1071 (26.2%)	466 (27.9%)	605 (25.0%)	
Xi’an City	891 (21.8%)	321 (19.2%)	570 (23.6%)	
Qishan County	1096 (26.8%)	454 (27.2%)	642 (26.5%)	
Hu County	1030 (25.2%)	428 (25.6%)	602 (24.9%)	
Smoking status				<0.001
Non-smoker	2937 (71.8%)	535 (32.1%)	2402 (99.3%)	
Pre-smoker	135 (3.3%)	134 (8.0%)	1 (0.0%)	
Current-smoker	1016 (24.9%)	1000 (59.9%)	16 (0.7%)	
Alcohol consumption				<0.001
Non-drinker	3108 (76.0%)	787 (47.2%)	232 1(95.9%)	
Pre-drinker	55 (1.4%)	52 (3.1%)	3 (0.1%)	
Current-drinker	925 (22.6%)	830 (49.7%)	95 (3.9%)	
Education				<0.001
Illiterate	163 (4.0%)	18 (1.1%)	145 (6.0%)	
Elementary school	323 (7.9%)	132 (7.9%)	191 (7.9%)	
Junior high school	1432 (35.0%)	610 (36.5%)	822 (34.0%)	
High school	966 (23.6%)	415 (24.9%)	551 (22.8%)	
Bachelor degree	1108 (27.1%)	458 (27.4%)	650 (26.9%)	
Graduate or above	96 (2.4%)	36 (2.2%)	60 (2.4%)	


*P value from students’*t*-test or Chi-square analysis; BMI, body mass index; WC, waist circumstance; HC, hip circumstance; WHR, waist-to-hip ratio; WHtR, waist-to-height ratio; FM%, fat mass percentage; LM%, lean mass percentage; VFR, visceral fat rating; SBP, systolic blood pressure; DBP, diastolic blood pressure.*

### Age-Related Changes of BP and Body Composition Indices in Healthy Subjects

**FIGURE 1:**
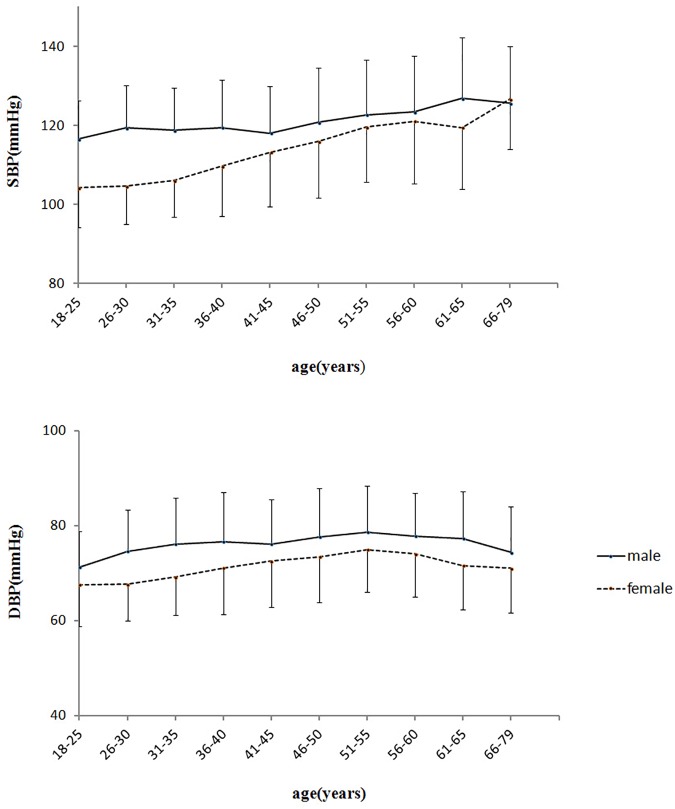
Age-related changes of blood pressure in healthy adults. SBP, systolic blood pressure; DBP, diastolic blood pressure.

In both genders, mean SBP levels maintained an increasing trend. From the age ranges of 18–25 years to 66–79 years, SBP increased by 9 and 22 mmHg in males and females, respectively. Mean DBP showed an inverted U-shape across the age span, with the levels increasing until the age of 55 and then declined. The difference of DBP between the peak age range of 51–55 years (79/75 mmHg) and the trough at age of 18–25 years (71/68 mmHg) was 8 and 7 mmHg for males and females, respectively (Figure 1, Table 1, and Supplementary Table S1).

All body composition indices changed with increasing ages in both genders (*P* < 0.001) (Supplementary Table S1). In males, height declined with age, while weight, BMI, WC, HC, WHR, WHtR, FM%, and VFR increased first and then declined, reaching a peak at 46–50 years for BMI, 65–70 years for VFR, and 51–55 years for the other indices. In females, height also declined with age, while weight, BMI, WC, HC, WHR, WHtR, FM%, and VFR all increased with age, reaching a peak at 51–55 years for weight, 65–70 years for VFR, and 61–65 years for the other indices. LM% declined with age, but showed a slightly increase after age of 50 and 70 years in males and females, respectively.

### BP Is Closely Correlated With Body Composition Indices in Healthy Subjects in Each Age Group

We found all adiposity-related indices, including general obesity index of BMI and FM%, and central obesity index of WHR, WHtR and VFR, were positively correlated with SBP and DBP in both genders. LM% was negatively correlated with SBP and DBP. Among all the indices, while BMI had the highest *r*-value of correlation with SBP in both males (*r* = 0.296, *p* < 0.001) and females (*r* = 0.237, *p* < 0.001), and FM% had the highest *r*-value of correlation with DBP in males (*r* = 0.351, *p* < 0.001) and females(*r* = 0.277, *p* < 0.001), the relationships with BP were similar across most of the body composition indices (Table 2).

**Table 2:** Partial Pearson Correlation between blood pressure and body composition indices in the subjects.

	SBP				DBP			
		
	male		female		male		female	
				
	*r*	*p*	*r*	*p*	*r*	*p*	*r*	*p*
BMI	0.296	<0.001	0.237	<0.001	0.350	<0.001	0.273	<0.001
WHR	0.221	<0.001	0.156	<0.001	0.290	<0.001	0.168	<0.001
WHtR	0.249	<0.001	0.199	<0.001	0.317	<0.001	0.228	<0.001
FM%	0.294	<0.001	0.232	<0.001	0.351	<0.001	0.277	<0.001
LM%	-0.294	<0.001	-0.229	<0.001	-0.350	<0.001	-0.275	<0.001
VFR	0.284	<0.001	0.231	<0.001	0.330	<0.001	0.270	0.001


*P was adjusted for age, education, smoking status, alcohol consumption, and residential location. BMI, body mass index; WHR, waist-to-hip ratio; WHtR, waist-to-height ratio; FM%, fat mass percentage; LM%, lean mass percentage; VFR, visceral fat rating; SBP, systolic blood pressure; DBP, diastolic blood pressure.*

**FIGURE 2:**
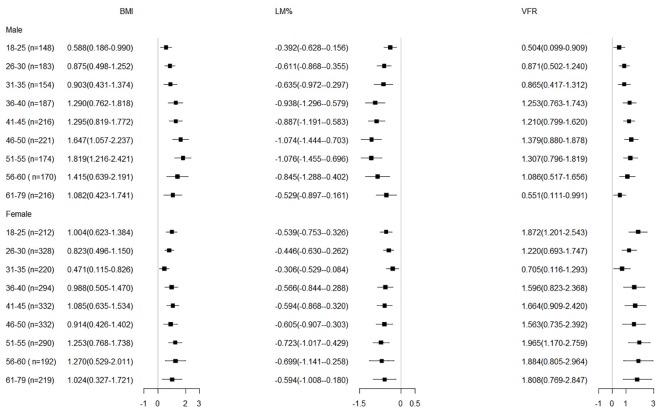
Adjusted regression coefficients of body composition indices by SBP in the subjects b (95% CI) was obtained from multiple linear regression analysis, adjusted for education, smoking and drinking status, and residential location. BMI, body mass index; LM%, lean mass percentage; VFR, visceral fat rating; SBP, systolic blood pressure.

**FIGURE 3:**
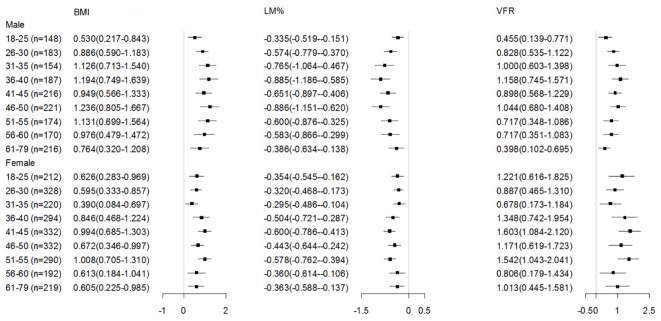
Adjusted regression coefficients of body composition indices by DBP in the subjects b (95% CI)was obtained from multiple linear regression analysis, adjusted for education, smoking and drinking status, and residential location; BMI, body mass index; LM%, lean mass percentage; VFR, visceral fat rating; SBP, systolic blood pressure.

To better understand the factors which may influence this age-related trend of BP increases, we analyzed the association of BP and body composition indices in each age group. We chose three representative indices, including BMI, VFR and LM%, which represent the status of general obesity, central obesity, and muscle mass, respectively. We found that both SBP and DBP were closely associated with BMI, LM%, and VFR in each age group, and the association between BP and body composition indices was weaker in the elderly compared to the younger ages (Figures 2, 3).

## Discussion

In this study, we demonstrated that mean SBP showed an age-related increase and mean DBP showed an inverted U-shape across the age span, and this trend was closely associated with the age-related body composition changes. Furthermore, we found that the association between BP and body composition indices was weaker in the elderly compared to the younger subjects. To the best of our knowledge, there has not been another study reporting such an age-related trend of BP closely correlated with body composition and tracks the entire life span of healthy subjects.

The association between body weight and BP has been well documented in both children and adults by some epidemiological studies (Brion et al., 2007; Dua et al., 2014; Dong et al., 2018). Weight gain was suggested to be strongly associated with BP increase (Ferriss et al., 2006; Middlemiss and McEniery, 2016). As demonstrated in our study, all measures of general obesity (BMI, FM%), central obesity (WHR, WHtR, and VFR) and LM% were correlated to BP at the whole population level, and among them the relationships with BP were similar across most of the body composition indices. As to which variable is the strongest predictor of BP, there are different opinions. Some studies suggested that general adiposity was more strongly correlated with BP (Wang et al., 2008; Chen X. et al., 2015), while other studies suggested central or visceral adiposity was more strongly correlated with BP than general adiposity (Ho et al., 2001; von Eyben et al., 2003; Wildman et al., 2005; Bennasar-Veny et al., 2013; Chandra et al., 2014; Abraham et al., 2015; Wang et al., 2015). Some studies demonstrated that after further adjusting for general adiposity, central adiposity turned out to not be strongly associated with BP (Ishizaka et al., 2009; Chen X. et al., 2015), indicating that central obesity was highly correlated with general obesity. This discrepancy may be due to the participants of all of these studies varied in age, race, and lifestyle. In this study, we didn’t find significant differences between these two kinds of obesity indices, as a low *r*-value between a predictor variable and BP might as well be due to the error term associated with the predictor variable as the absence of a biological relationship. Therefore, mechanism about this discrepancy may be in need of further investigation.

To examine whether body composition was a factor influencing BP throughout the whole adult age span, we further analyzed the association of BP with BMI, LM% and VFR in each specific age-group (at 5-year ranges). After adjustment for education level, smoking status, alcohol consumption and residential location, BMI and VFR were positively associated with BP in each age group, suggesting that adiposity was an important risk factor for the increased BP, whereas LM% was negatively associated with BP, the latter indicating its protective effect on BP, similar to the result reported by Park et al. (Kim et al., 2011; Park and Yoon, 2013). Thus, our results support the strategy of weight control for reducing BP as secondary prevention of cardiovascular events, which is in accordance with Dorresteijn et al. (2012). The correlation between BP and all these three measures (BMI, LM%, and VFR) was weaker in the elderly than younger adults. Kotsis et al. (2015) reported that while increased BMI was positively associated with increased BP, a 10-20% of the BP increase could be attributed to the increase of BMI, suggesting that obesity is only one of the contributors to BP increase. Thus, as demonstrated by our study, we may infer that factors associated with increased BP may be more complicated in the elderly compared to the younger age groups.

The strength of this study was, first, we divided the population into 10 age groups, from the age ranges of 18–25 years to 66–79 years, therefore could precisely assess and compare the contribution of body composition to BP in each age group. Second, we excluded subjects with pre-existing hypertension and systematic chronic diseases, which might excluded the influence of the drug therapy on the blood pressure, so leading the results more precise. This study has some limitations. First, information about education, smoking status and alcohol consumption was self-reported, which might have bias. Second, the sample size of this study is relatively small, especially for the elderly (66–79 years), we did not analyze by stratification of that large age range. Third, it is infeasible to collect the information of the dead in the practice due to the layout of the study. Fourth, since we measured BP once in the clinic office, there may be “white coat” hypertension. Fifth, Shanxi province is located in north-west of middle China, which may not represent the national population and limited applicability to the wider population. Additionally, although different age groups were involved in this study, they may not represent the growth patterns of BP and adiposity which can only be addressed in a longitudinal study.

In this study, we reported that BP showed an age-related increase trend, and this trend was closely associated with the age-related body fat or muscle mass changes. Our findings indicated that maintain the total body fat in a favorable range and appropriately increase the body muscle mass is a strategy to reduce the occurrence of cardiovascular event by decreasing the risk of hypertension through the whole adult life.

## Author Contributions

WL and YH carried out the experimental design, participated in the data analysis and drafted the manuscript. They contributed equally to this study and share first authorship. LX and XY participated in the experimental design and the review of the manuscript. FL, JM, ZH, and YL participated in the data collection and reviewed the manuscript. DL and JJ participated in the literature search and reviewed the manuscript. GS and CL contributed to the experimental design and review of this manuscript. All authors read and approved the final manuscript.

## Conflict of Interest Statement

The authors declare that the research was conducted in the absence of any commercial or financial relationships that could be construed as a potential conflict of interest.

## Footnotes

1 http://www.r-project.org/
