# Supplementary material for: Association of Age-Related Trends in Blood Pressure and Body Composition Indices in Healthy Adults
Source: Front Physiol. 2018 Nov 26;9:1574. doi: 10.3389/fphys.2018.01574 (PMC6275465; doi:10.3389/fphys.2018.01574)
Supplement: Supplementary file 1 [file Table_1.docx]

Table S1. Anthropometric characteristics stratified by age in the healthy subjects

| **Age Group** | **18-25** | **26-30** | **31-35** | **36-40** | **41-45** | **46-50** | **51-55** | **56-60** | **61-65** | **66-79** | ***P* value** |
| --- | --- | --- | --- | --- | --- | --- | --- | --- | --- | --- | --- |
| Male |  |  |  |  |  |  |  |  |  |  |  |
| N=1669 | 148 | 183 | 154 | 187 | 216 | 221 | 174 | 170 | 114 | 102 |  |
| Age(years) | 20.74±2.18 | 27.72±1.62 | 32.91±1.53 | 37.93±1.42 | 42.91±1.52 | 48.01±1.51 | 52.98±1.48 | 57.84±1.44 | 62.95±1.27 | 70.05±3.54 | <0.001 |
| Height(cm) | 172.36±6.08 | 171.99±6.42 | 169.90±5.50 | 169.43±5.64 | 169.62±5.19 | 167.63±5.52 | 168.34±5.93 | 167.61±5.83 | 167.36±5.88 | 165.16±6.11 | <0.001 |
| Weight(kg) | 65.11±12.50 | 70.68±13.01 | 69.34±11.63 | 68.92±9.89 | 68.56±10.43 | 67.43±9.11 | 68.06±10.84 | 66.27±9.46 | 64.89±9.75 | 61.87±9.63 | <0.001 |
| BMI(kg/m^2^) | 21.89±3.93 | 23.87±4.03 | 23.98±3.67 | 23.99±3.15 | 23.80±3.20 | 24.00±2.91 | 23.93±3.16 | 23.54±2.81 | 23.09±2.79 | 22.64±3.06 | <0.001 |
| WC(cm) | 80.43±11.79 | 87.46±11.2 | 87.27±9.35 | 87.87±8.84 | 86.98±8.98 | 88.19±8.40 | 89.70±8.81 | 88.27±8.45 | 87.22±8.87 | 85.21±11.97 | <0.001 |
| HC(cm) | 96.00±6.95 | 98.66±6.34 | 97.81±5.91 | 97.78±5.08 | 97.10±5.48 | 97.25±4.87 | 97.61±6.12 | 96.31±4.58 | 96.21±5.14 | 95.43±5.88 | <0.001 |
| WHR | 0.83±0.07 | 0.88±0.07 | 0.89±0.06 | 0.90±0.06 | 0.89±0.06 | 0.91±0.06 | 0.92±0.05 | 0.92±0.06 | 0.91±0.06 | 0.89±0.10 | <0.001 |
| WHtR | 0.47±0.07 | 0.51±0.06 | 0.51±0.05 | 0.52±0.05 | 0.51±0.05 | 0.52±0.05 | 0.53±0.05 | 0.53±0.05 | 0.52±0.05 | 0.52±0.07 | <0.001 |
| FM% | 15.52±6.92 | 19.87±6.22 | 20.10±5.45 | 20.58±4.90 | 20.20±5.26 | 20.66±4.95 | 21.00±5.36 | 20.12±5.16 | 19.01±5.37 | 18.47±5.94 | <0.001 |
| LM(%) | 80.07±6.56 | 75.95±5.89 | 75.75±5.16 | 75.29±4.64 | 75.66±4.97 | 75.20±4.69 | 74.89±5.09 | 75.71±4.90 | 76.77±5.09 | 77.27±5.61 | <0.001 |
| VFR | 4.59±3.88 | 7.66±4.10 | 8.51±3.87 | 9.44±3.38 | 9.93±3.68 | 10.76±3.49 | 11.38±3.90 | 11.55±3.79 | 11.66±4.24 | 11.87±4.74 | <0.001 |
| SBP(mm Hg) | 116.50±9.68 | 119.3±10.76 | 118.79±10.66 | 119.44±12.09 | 118.05±11.87 | 120.81±13.76 | 122.64±13.91 | 123.44±14.09 | 126.77±15.45 | 125.65±14.27 | <0.001 |
| DBP(mm Hg) | 71.21±7.61 | 74.55±8.81 | 76.08±9.71 | 76.63±10.40 | 76.03±9.45 | 77.63±10.16 | 78.62±9.76 | 77.73±9.07 | 77.31±9.87 | 74.33±9.58 | <0.001 |
| Female |  |  |  |  |  |  |  |  |  |  |  |
| N=2419 | 212 | 328 | 220 | 294 | 332 | 332 | 290 | 192 | 118 | n=101 |  |
| Age(years) | 21.48±2.11 | 27.61±1.70 | 32.99±1.43 | 38.04±1.33 | 43.08±1.46 | 47.98±1.42 | 52.71±1.45 | 57.80±1.46 | 62.61±1.35 | 70.55±3.45 | <0.001 |
| Height(cm) | 159.10±5.21 | 158.60±5.25 | 158.27±5.41 | 157.84±5.40 | 156.81±5.08 | 156.71±5.13 | 156.51±5.10 | 154.77±5.16 | 155.15±5.80 | 153.19±5.29 | <0.001 |
| Weight(kg) | 52.16±9.24 | 53.49±8.36 | 56.29±9.14 | 56.30±7.93 | 57.44±8.60 | 57.89±8.53 | 58.57±8.79 | 56.84±8.17 | 58.01±8.64 | 54.72±8.94 | <0.001 |
| BMI(kg/m^2^) | 20.61±3.43 | 21.26±3.17 | 22.48±3.47 | 22.58±2.97 | 23.35±3.25 | 23.56±3.21 | 23.89±3.27 | 23.75±3.14 | 24.02±3.06 | 23.29±3.48 | <0.001 |
| WC(cm) | 73.12±9.80 | 74.81±8.11 | 79.30±9.63 | 78.71±8.77 | 80.85±9.90 | 82.30±8.96 | 84.57±8.64 | 83.67±9.85 | 86.83±8.74 | 85.59±10.77 | <0.001 |
| HC(cm) | 91.18±6.71 | 92.09±5.75 | 94.03±6.43 | 93.72±6.05 | 94.51±5.84 | 94.87±5.65 | 95.47±6.09 | 94.05±6.24 | 96.00±6.23 | 95.33±5.49 | <0.001 |
| WHR | 0.80±0.07 | 0.81±0.06 | 0.84±0.06 | 0.84±0.06 | 0.85±0.07 | 0.87±0.06 | 0.88±0.06 | 0.89±0.07 | 0.90±0.06 | 0.90±0.08 | <0.001 |
| WHtR | 0.46±0.06 | 0.47±0.05 | 0.50±0.06 | 0.50±0.06 | 0.52±0.06 | 0.52±0.06 | 0.54±0.06 | 0.54±0.06 | 0.56±0.06 | 0.56±0.07 | <0.001 |
| FM% | 25.78±6.47 | 27.38±5.90 | 29.96±5.73 | 30.15±5.39 | 31.25±5.59 | 31.98±5.39 | 32.79±5.61 | 32.70±5.46 | 33.32±5.19 | 31.90±6.26 | <0.001 |
| LM(%) | 70.06±6.16 | 68.49±5.66 | 66.02±5.48 | 65.84±5.16 | 64.80±5.33 | 64.11±5.16 | 63.36±5.38 | 63.57±5.27 | 62.89±4.98 | 64.34±6.03 | <0.001 |
| VFR | 2.68±1.93 | 3.37±1.98 | 4.50±2.10 | 4.84±1.85 | 5.63±1.94 | 6.11±1.89 | 6.59±2.00 | 6.91±2.14 | 7.32±1.79 | 7.37±2.59 | <0.001 |
| SBP(mm Hg) | 104.21±10.10 | 104.64±9.68 | 106.03±9.24 | 109.66±12.69 | 113.24±13.87 | 115.90±14.21 | 119.57±13.92 | 121.11±15.90 | 119.46±15.61 | 126.59±12.69 | <0.001 |
| DBP(mm Hg) | 67.56±8.85 | 67.63±7.75 | 69.11±8.00 | 71.11±9.90 | 72.47±9.66 | 73.43±9.61 | 74.96±8.94 | 74.01±9.06 | 71.61±9.39 | 71.01±9.32 | <0.001 |

BMI, body mass index; WC, waist circumstance; HC, hip circumstance; WHR, waist-to-hip ratio; WHtR, waist-to-height ratio; FM%, fat mass percentage; LM%, lean mass percentage; VFR, visceral fat rating; SBP, systolic blood pressure; DBP, diastolic blood pressure
